# Multi-institutional Development and Implementation of an Internal Medicine-Pediatrics Acting Sub-internship

**DOI:** 10.7759/cureus.115263

**Published:** 2026-08-26

**Authors:** Patrick G Smith, Ashmita Chatterjee, Casey Gradick, William Rearick, Robert Campbell, Richard Hobbs, Benjamin M Drum

**Affiliations:** 1 Internal Medicine-Pediatrics, University of Utah, Salt Lake City, USA; 2 Internal Medicine-Pediatrics, University of North Carolina at Chapel Hill, Chapel Hill, USA

**Keywords:** acting internship, curriculum implementation, med-peds, residency applicant, sub-internship

## Abstract

Background: Medical students interested in combined Internal Medicine-Pediatrics (Med-Peds) training traditionally complete separate sub-internships in Internal Medicine and Pediatrics. This fragmented approach often limits access to Med-Peds-specific mentorship, integrated clinical training, and specialty-aligned letters of recommendation.

Objective: To describe the development, implementation, feasibility, and preliminary outcomes of two institution-specific integrated Med-Peds sub-internships.

Methodology: The University of North Carolina (UNC) and the University of Utah independently developed four-week Med-Peds sub-internships led by Med-Peds-trained faculty. UNC implemented a Med-Peds hospitalist acting internship, whereas Utah developed a core sub-internship consisting of two-week inpatient rotations on internal medicine and pediatrics services, both supervised by Med-Peds attendings. Educational outcomes, student evaluations, access to letters of recommendation, and residency match data were assessed.

Results: At UNC, 182 students completed the Med-Peds sub-internship. Following implementation, the UNC Med-Peds match rate among the entire graduating class increased from 3.3% to 4.6% (*P* = 0.005). UNC course evaluations were consistently high (mean overall rating 4.68/5; faculty effectiveness 3.92/4). At the University of Utah, 10 students completed the course, and all rated the experience as *excellent*, citing autonomy within a supportive learning environment. Additionally, access to Med-Peds-specific letters of recommendation increased from 33% to 100%, exceeding national averages (approximately 60%).

Conclusions: Integrated Med-Peds sub-internships address a longstanding curricular gap by providing cohesive clinical exposure, dedicated mentorship, and specialty-specific career development. These models are feasible, adaptable, and may positively influence student satisfaction and Med-Peds match outcomes at institutions with dedicated Med-Peds faculty.

## Introduction

Throughout medical school, several factors contribute to both a successful residency application and the development of a confident applicant. For most students, the process begins with selecting a specialty-a decision influenced by personality, clinical interests, salary, exposure, and lifestyle considerations [1,2]. The core clerkships (internal medicine, psychiatry, surgery, pediatrics, etc.) provide foundational exposure for medical students; however, certain surgical subspecialties and combined programs - such as, but not limited to, Internal Medicine-Psychiatry, Urology, Ophthalmology, Internal Medicine-Emergency Medicine, Triple Board, and Internal Medicine-Pediatrics (Med-Peds) - are often not included in standard rotations. Consequently, students interested in these fields may have limited opportunities for early exposure and clinical experience. To bridge this gap, some institutions have implemented earlier clinical experiences [3]. In this paper, the introduction of a specialty-specific sub-internship is examined as an intervention designed to support students pursuing a combined residency in Internal Med-Peds.

Sub-internships play a pivotal role in the residency application process. They allow students to function at the level of an intern while strengthening clinical competence, practice-based learning, interprofessional collaboration, and communication skills [3]. Direct supervision within a chosen specialty fosters mentorship, facilitates specialty-specific letters of recommendation, and helps students confirm or reconsider their career choice [4,5]. Moreover, these experiences cultivate the skills residency faculty expect of incoming interns [6].

Because sub-internships frequently coincide with the residency application timeline, they also serve as a critical platform for securing strong letters of recommendation. In combined specialties such as Med-Peds, where specialty-specific mentorship may be less accessible due to the rarity of specialty-specific faculty, integrated sub-internships provide a structured opportunity to build relationships, demonstrate clinical ability, and obtain meaningful, field-specific endorsements.

Students pursuing Med-Peds residency training traditionally complete categorical sub-internships in either Internal Medicine, Pediatrics, or both. Although this structure provides exposure to each discipline, it often lacks integration and may limit meaningful interaction with Med-Peds faculty. As a result, students can experience fragmented training, reduced access to specialty-specific mentorship, and difficulty obtaining Med-Peds-focused letters of recommendation - factors that may hinder both career exploration and residency application success. In response to these challenges, the University of North Carolina (UNC) and the University of Utah independently developed integrated Med-Peds sub-internships to better support students interested in the specialty.

The primary objective of this multi-institutional study was to describe the development and implementation of two integrated Internal Med-Peds sub-internships at the UNC and the University of Utah. Secondary objectives were to: (1) evaluate student satisfaction and perceived educational value of the integrated rotations; (2) assess the impact of these curricula on access to Med-Peds-specific letters of recommendation at the University of Utah; (3) examine changes in the institutional Med-Peds residency match rate following implementation of the acting internship (AI) at UNC; and (4) determine the feasibility and adaptability of integrated Med-Peds sub-internships across institutions with varying levels of Med-Peds faculty resources.

## Materials and methods

Setting and participants

The UNC School of Medicine currently enrolls 204 students annually. UNC’s Med-Peds residency program accepts six to seven residents per year. UNC Hospitals is a public, tertiary-care, academic healthcare system. 

The Spencer Fox Eccles School of Medicine at the University of Utah currently enrolls 127 students annually. The University of Utah Med-Peds residency program accepts three residents per year. The University of Utah Hospital system is a public, tertiary-care, academic healthcare system.

Intervention

The Med-Peds sub-internships at both institutions were designed to provide fourth-year medical students with an immersive clinical experience that integrates internal medicine and pediatrics while promoting specialty-specific mentorship, clinical autonomy, and exposure to the breadth of Med-Peds practice. The curricula were developed to address limited opportunities for specialty-specific clinical experience during traditional medical school training.

The primary educational objectives were to enable students to: function at the level of a supervised intern on both pediatric and adult inpatient services; develop clinical decision-making skills across the lifespan; experience integrated Med-Peds practice; establish mentorship relationships with Med-Peds faculty and residents; and better prepare for residency applications and career decision-making.

UNC

In 2012, UNC implemented a four-week Med-Peds Combined Hospitalist Sub-Internship (also known as an AI). The departments of Internal Medicine and Pediatrics officially approved the course, which is open to fourth-year medical students from UNC and visiting students, with priority given to UNC students. Two to three students may enroll per clinical block.

During the rotation, students spend two weeks each in Pediatrics and Internal Medicine, assuming the responsibilities of an intern. From 2012 to 2015, students worked exclusively with hospitalists in both Internal Medicine and Pediatrics. In 2015, the pediatric portion of the curriculum was modified following implementation of a traditional resident teaching service, allowing students to participate on multidisciplinary inpatient teams alongside residents and third-year medical students.

While completing this rotation, students are immersed in the daily life of the respective residency programs, participating in morning report, journal club, noon conference, and Grand Rounds, as well as Med-Peds-specific residency conferences.

Course directors create student schedules, pairing them with Med-Peds-trained attendings and residents when possible. A sample of this schedule is provided in the supplemental material (Appendix A). Students receive standard orientation materials, rotation objectives, and schedules prior to the start of the sub-internship. During their rotation, students work with two to three attendings in each department throughout the course and have the opportunity to spend one half-day in the Med-Peds resident clinic. At the end of the rotation, course directors gather student feedback from attendings and aggregate comments to assign a grade (Fail, Pass, High Pass, or Honors). 

Utah

The University of Utah implemented the Med-Peds sub-internship starting in 2023. This course was approved by the Curriculum Committee and fulfills the student requirement to complete a core sub-internship. Similar to UNC, the course is open to fourth-year medical students from Utah and visiting students, with priority granted to Utah students. All scheduling and evaluations are organized by the Med-Peds coordinator to offset any additional burden on the categorical Internal Medicine or Pediatric programs.

At the University of Utah, there are a total of 17 Med-Peds faculty members (3 practicing only pediatrics, 7 practicing only internal medicine, and 7 practicing both). Consequently, the course is offered during only one four-week block in the summer. Whenever possible, students are paired with board-certified Med-Peds faculty who serve as both clinical supervisors and mentors throughout the rotation.

During the rotation, students spend two weeks each in Pediatrics and Internal Medicine, assuming the responsibilities of an intern. An example of the schedule is provided in the supplemental material (Appendix B). During Internal Medicine, two students work directly with one Med-Peds attending physician. During Pediatrics, students work on separate resident teams led by a Med-Peds attending physician and with Med-Peds residents, as able.

Similar to UNC, students are immersed in the daily life of the respective residency programs, participating in morning report, journal club, noon conference, and Grand Rounds. Students also lead the Med-Peds resident journal club and attend a half-day Med-Peds clinic tailored to their career interests, including transition clinics such as spina bifida and rheumatology clinics, where Med-Peds faculty help prepare pediatric patients for transition to adult care. At the end of the rotation, course directors gather student feedback from attendings and aggregate comments to assign a grade (Fail, Pass, High Pass, or Honors). 

Both curricula emphasized clinical autonomy, integrated care across age groups, and longitudinal mentorship. 

Analysis

UNC

The potential impact of the course on residency placement was evaluated using course rosters and publicly available match data on residency location and discipline at the national level. The number of applicants matching into Med-Peds was also tracked.

Students in the Med-Peds AI completed course and faculty evaluations between 2012 and 2025. For each component, the mean was calculated. The study was reviewed and approved by the UNC Institutional Review Board.

Utah

Students completed course and faculty evaluations, with individual components rated on a Likert scale (Appendix C). For each component, the mean was calculated. Students were also surveyed six months after course completion, with individual components rated on a Likert scale (Appendix D). On the six-month follow-up survey, students were asked how many letters of recommendation were from Med-Peds-trained faculty. The study was reviewed and deemed exempt by the University of Utah Institutional Review Board.

At both institutions, surveys included the options of Strongly Agree, Agree, Neutral, Disagree, and Strongly Disagree and were scored on a Likert scale.

## Results

**Figure 1:**
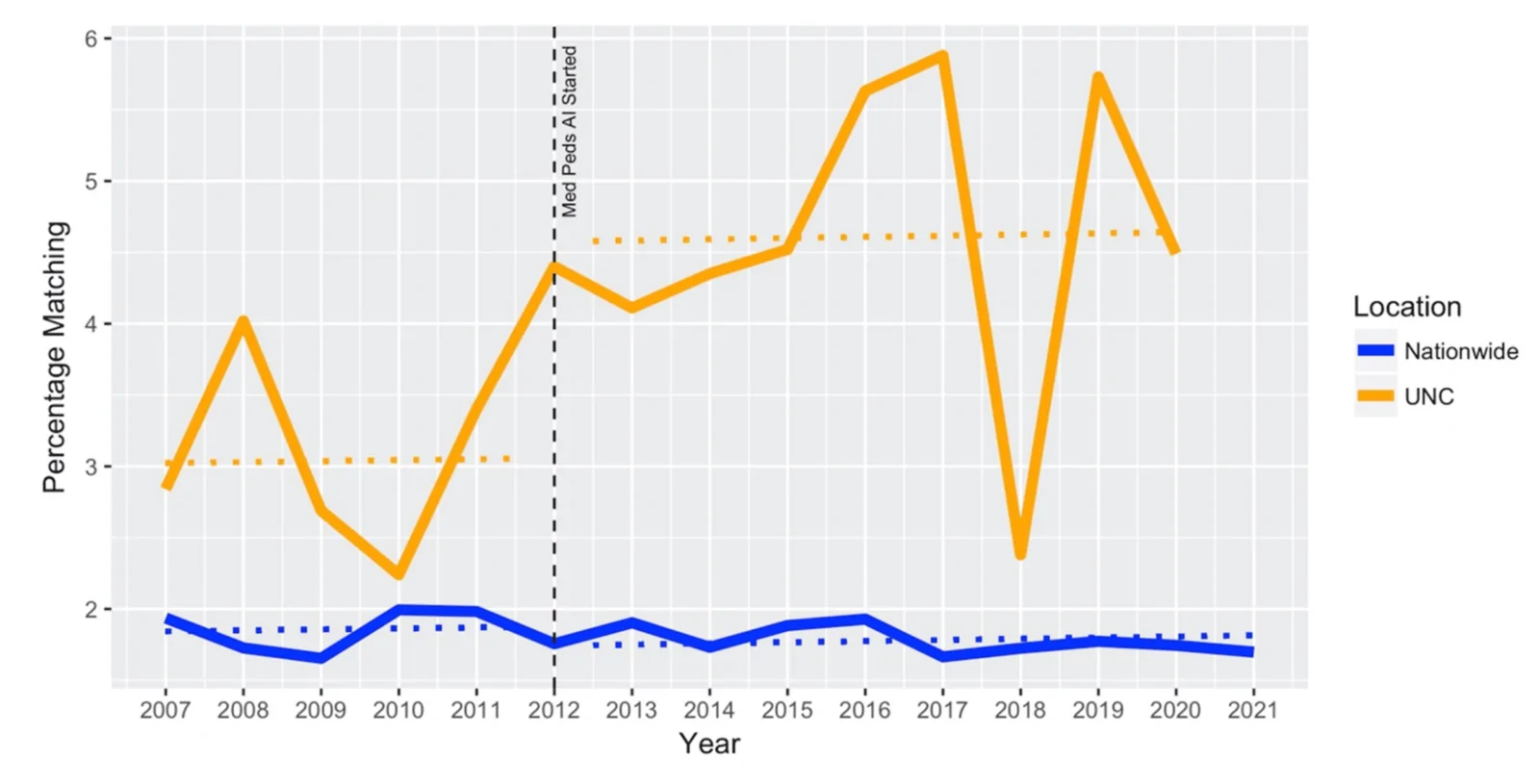
UNC match data. Comparison of UNC Med-Peds match rate among the graduating class (orange line) vs. the national average (blue line). The horizontal dashed lines represent the average across the years, with UNC having two averages split by the vertical dashed line (inception of AI). The image was generated in Python using Matplotlib. AI, Acting Intern; UNC, University of North Carolina; Med-Peds, Medicine-Pediatrics

At UNC, 182 students completed the Med-Peds AI. Survey scores indicate that students generally had a positive experience after completing the rotation (Table 1). Notably, student evaluations demonstrated high satisfaction, with an average overall course rating of 4.68 out of 5. Following implementation, the proportion of UNC students matching into Med-Peds increased from 3.3% among graduates between 2007 and 2012 to 4.6% among graduates between 2013 and 2020 (*P* = 0.005, Figure 1). The raw data supporting these findings are available in the supplemental material (Appendices E-F).

**Table 1:** University of North Carolina composite Med-Peds hospitalist acting internship rotation evaluation, 2012-2025 (N = 182), 5-point Likert scale. *Students used a 5-point Likert scale with 1 = “Strongly Disagree,” 2 = “Disagree,” 3 = “Neither Disagree nor Agree,” 4 = “Agree,” and 5 = “Strongly Agree,” except the overall rating of the elective, for which students used a 5-point Likert scale with 1 = “Poor,” 2 = “Fair,” 3 = “Good,” 4 = “Very Good,” and 5 = “Excellent.” †From 2012 to 2025, multiple different evaluation questionnaires were used. Although all used Likert scales, the numerical scales differed. To normalize the data, scores are reported as percentages rather than absolute values. Additionally, some questions with slightly different wording but the same meaning (i.e., “This elective” vs. “This course”) were combined.

Questions used for evaluation^†^	Mean score*
This elective helped me develop skills in analyzing clinical or research problems	4.70
This elective helped me develop judgment in addressing research or patient management issues	4.80
Faculty gave appropriate feedback on my performance and progress	4.70
Clinical experience	
I was given the appropriate level of responsibility for patient care for a student.	4.60
The clinical supervision I received was appropriate.	4.70
Faculty promoted an environment conducive to learning.	4.75
Overall rating of this elective	4.70

**Figure 2:**
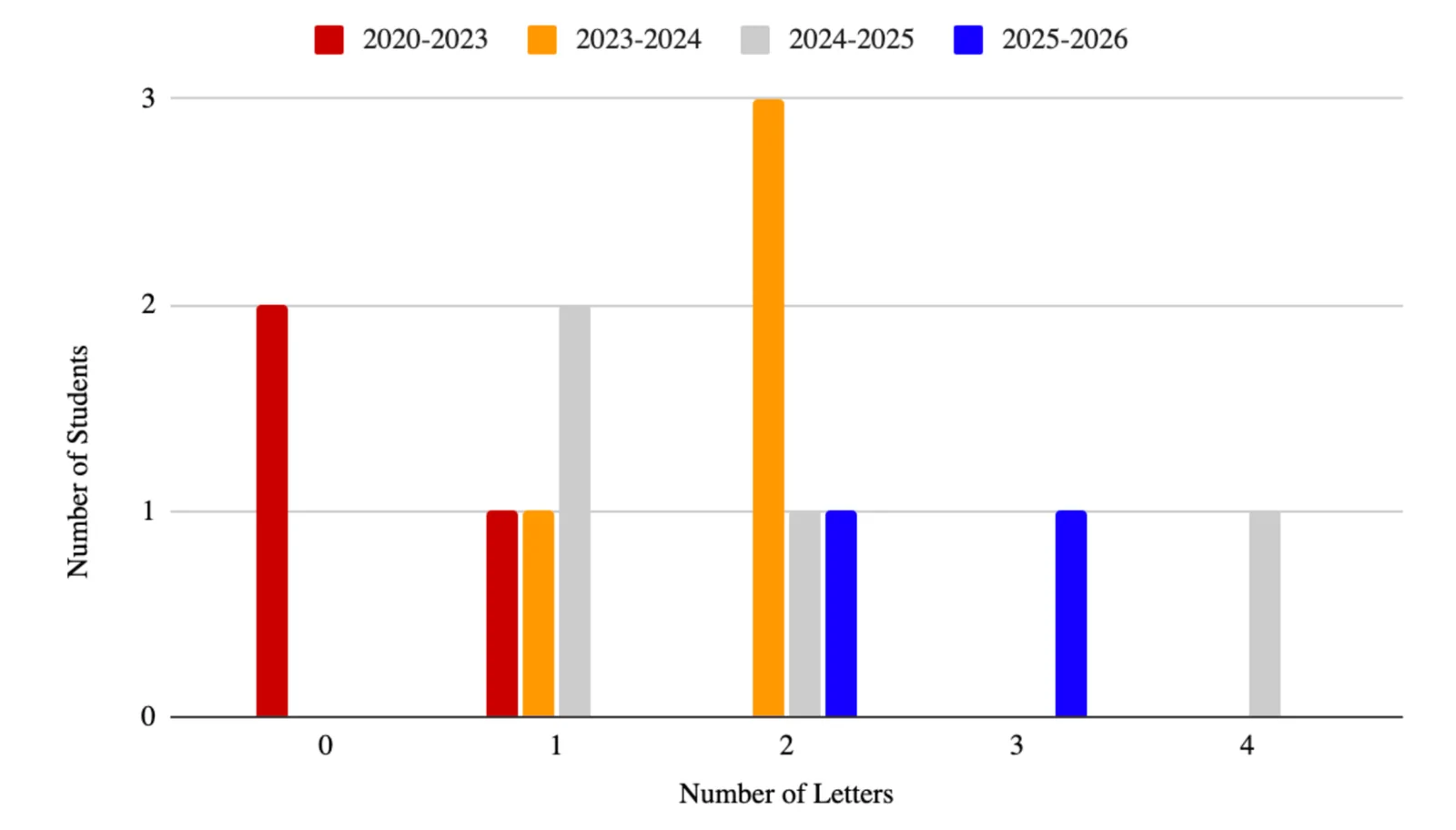
Letters of recommendation at the University of Utah. Number of Med-Peds-specific letters of recommendation for medical students completing sub-internships at the University of Utah. Data from 2020 to 2023 were combined because they predated the combined sub-internship.

In the newer core sub-internship at the University of Utah, 10 students completed the course, with most rating it as an *excellent* experience, with a score of 4.8 out of 5, highlighting the value of autonomy in a supportive learning environment. Overall, the survey results were reassuring and demonstrated the successful rollout of the rotation (Table 2). Additionally, the proportion of students receiving Med-Peds-specific letters of recommendation (defined as ≥1 letter) increased from 33% to 100% (national average, approximately 60%), reflecting improved access to specialty-aligned mentorship (Figure 2). Moreover, 7 of the 10 students had 2 or more letters from Med-Peds-specific faculty.

**Table 2:** University of Utah composite Med-Peds hospitalist acting internship rotation evaluation, 2023-2026 (N = 10), 5-point Likert scale. *Students used a 5-point Likert scale with 1 = “Strongly Disagree,” 2 = “Disagree,” 3 = “Neither Disagree nor Agree,” 4 = “Agree,” and 5 = “Strongly Agree.”

Questions used for evaluation	Mean score*	
At completion of rotation		
The patient-to-student ratio was conductive for learning.	4.90	
Faculty provided effective feedback	4.80	
I received useful formative feedback.	4.70	
The journal club presentation was useful.	4.60	
The transitions experience was useful.	4.40	
This course was of excellent quality.	4.80	
Six-month post sub-internship survey		
I wish I had completed one/both categorical sub-internships instead of this course.	2.10	
The additional med-peds experiences were valuable and should be included in this course.	4.60	
Working with med-peds faculty improved the quality of the course.	4.60	
This course helped me obtain relevant letters of recommendation for my residency application.	4.80	
This course helped clarify and/or affirm my career goals.	4.90	
I feel better prepared for residency as a result of taking this course.	4.90	
Overall, I’m glad I completed this course.	4.90	

## Discussion

There is limited literature examining the factors that contribute to a successful residency application. However, existing studies suggest that specialty-specific exposure and direct mentorship may play important roles in both application development and career decision-making [7-9]. The integrated Med-Peds sub-internships described here provide a structured clinical experience through a Med-Peds lens and may help address some of the challenges students face when seeking specialty-specific exposure. These programs provide mentorship opportunities, support informed specialty selection, and facilitate access to specialty-aligned letters of recommendation. Importantly, the two institutions represent different levels of Med-Peds hospitalist involvement, demonstrating that the structure of a Med-Peds sub-internship can be adapted to the clinical resources and faculty available at an individual institution.

The observed increase in Med-Peds match rates at UNC and high levels of student satisfaction at both institutions suggest that these rotations may offer educational and career-related benefits. Although outcomes were assessed only at UNC and the University of Utah in this study, other institutions, such as the University of Kansas City and Christiana Care, have developed Med-Peds sub-internship opportunities with similar goals, suggesting broader interest in this model [10,11]. In addition, Christiana Care offered a virtual Med-Peds elective during the COVID-19 pandemic. Over three years, five students completed the course each year. All students reported that the experience met or exceeded their expectations, and most identified Med-Peds as their top choice for residency at the time of application [12]. Together, these experiences provide preliminary support for the feasibility of both in-person and virtual Med-Peds-specific electives and suggest that such experiences may help students better understand the specialty and prepare for the residency application process.

An additional benefit to sub-internships is the fact that they may improve the likelihood of receiving residency interviews through strengthening of several application components valued by program directors. A national survey of residency program directors revealed that multiple applicant attributes, including letters of recommendation, interpersonal skills, scholarly work, and evidence of professional engagement, play important roles in determining which applicants are selected for interviews within a holistic review process [13]. Sub-internships provide an opportunity for applicants to demonstrate many of these qualities directly through clinical performance, teamwork, and mentorship interactions. Second, a study analyzing over 400,000 residency applications found that completing a sub-internship as a visiting student (away rotation) significantly increased the odds of receiving an interview invitation, exposing the potential value for students exploring outside programs besides their home institution [14]. Developing more rotations as described in this paper at other institutions may help applicants be exposed to more programs and match at a desired location. 

This study has several limitations. First, the findings are based on the experiences of two institutions and may not be generalizable to all Med-Peds training programs or other combined specialties. Second, the evaluation relied primarily on observational data and student self-report measures, which are subject to response bias and cannot establish causal relationships between participation in the integrated sub-internship and educational or residency application outcomes. In addition, the increase in the Med-Peds residency match rate observed at UNC should be interpreted cautiously. Because this analysis was observational and did not include a comparison group or adjustment for potential confounding factors, changes in match outcomes may have been influenced by concurrent institutional, applicant, or national trends rather than the sub-internship alone. Therefore, these findings should be considered preliminary and hypothesis-generating. 

## Conclusions

Medical students interested in Med-Peds often face challenges in obtaining specialty-specific clinical exposure, mentorship, and letters of recommendation. The Med-Peds sub-internships at UNC and the University of Utah were designed to address these gaps. At both institutions, students reported high levels of satisfaction with the rotations. Utah students also demonstrated increased access to Med-Peds-specific letters of recommendation, while UNC observed an increase in the proportion of students matching into Med-Peds. These findings suggest that integrated sub-internships may strengthen specialty identity, support informed career decision-making, and enhance preparation for the residency application process through specialty-specific mentorship, direct observation of clinical performance, and increased access to specialty-specific letters of recommendation. By fostering meaningful relationships with Med-Peds faculty and increasing exposure to the specialty, these experiences may also improve opportunities to obtain residency interviews and ultimately match into Med-Peds.

Future multi-institutional studies with longitudinal follow-up could better characterize the impact of these experiences on residency outcomes and career development. Nevertheless, our findings suggest that integrated Med-Peds sub-internships represent a feasible and adaptable educational model that may serve as a model for other Med-Peds programs and potentially other combined specialties.

## Figures and Tables

**Figure 3:**
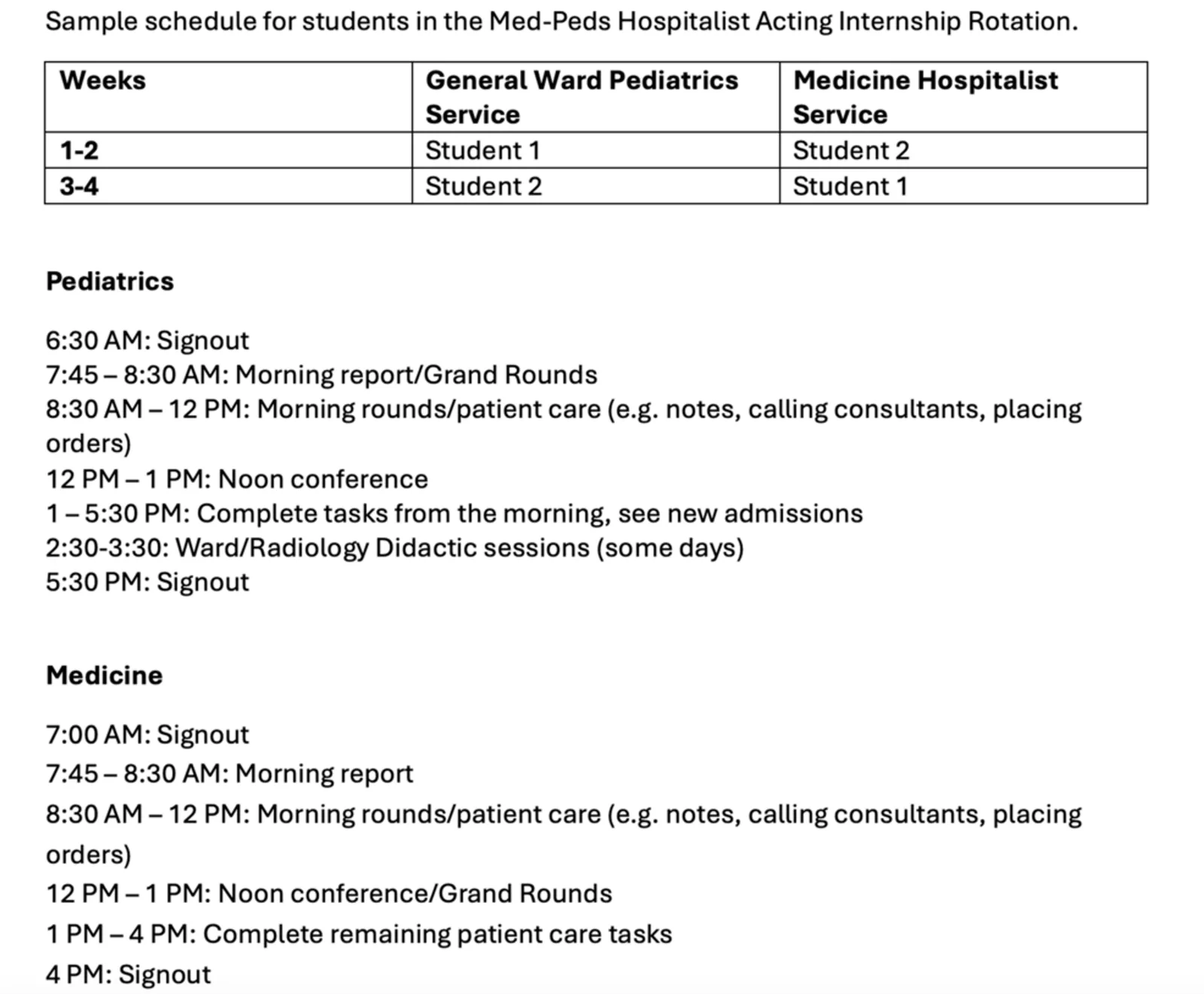
Sample Schedule for a Sub-Intern at the University of North Carolina.

**Figure 4:**
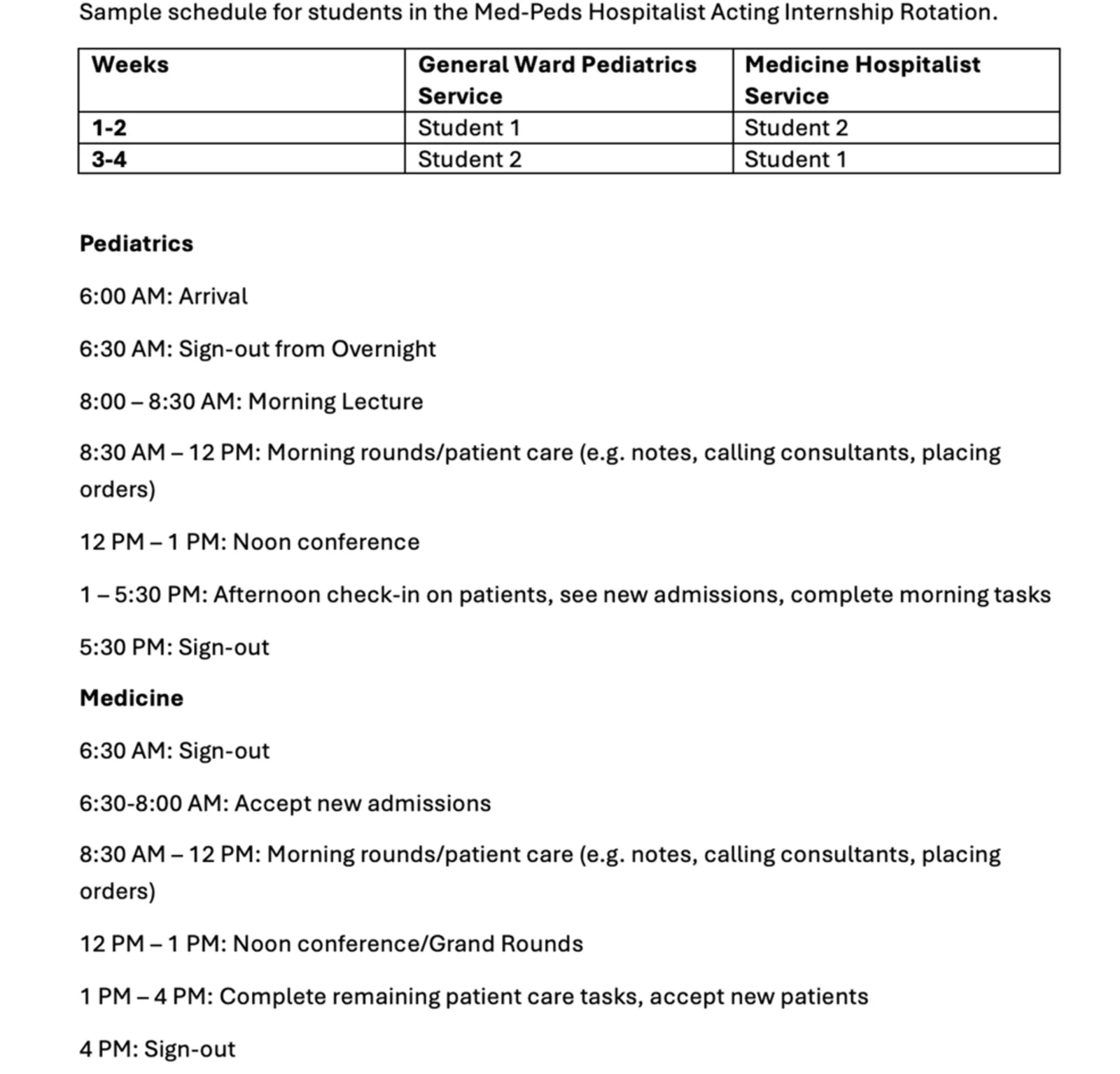
Sample Schedule for a Sub-Intern at the University of Utah.

**Figure 5:**
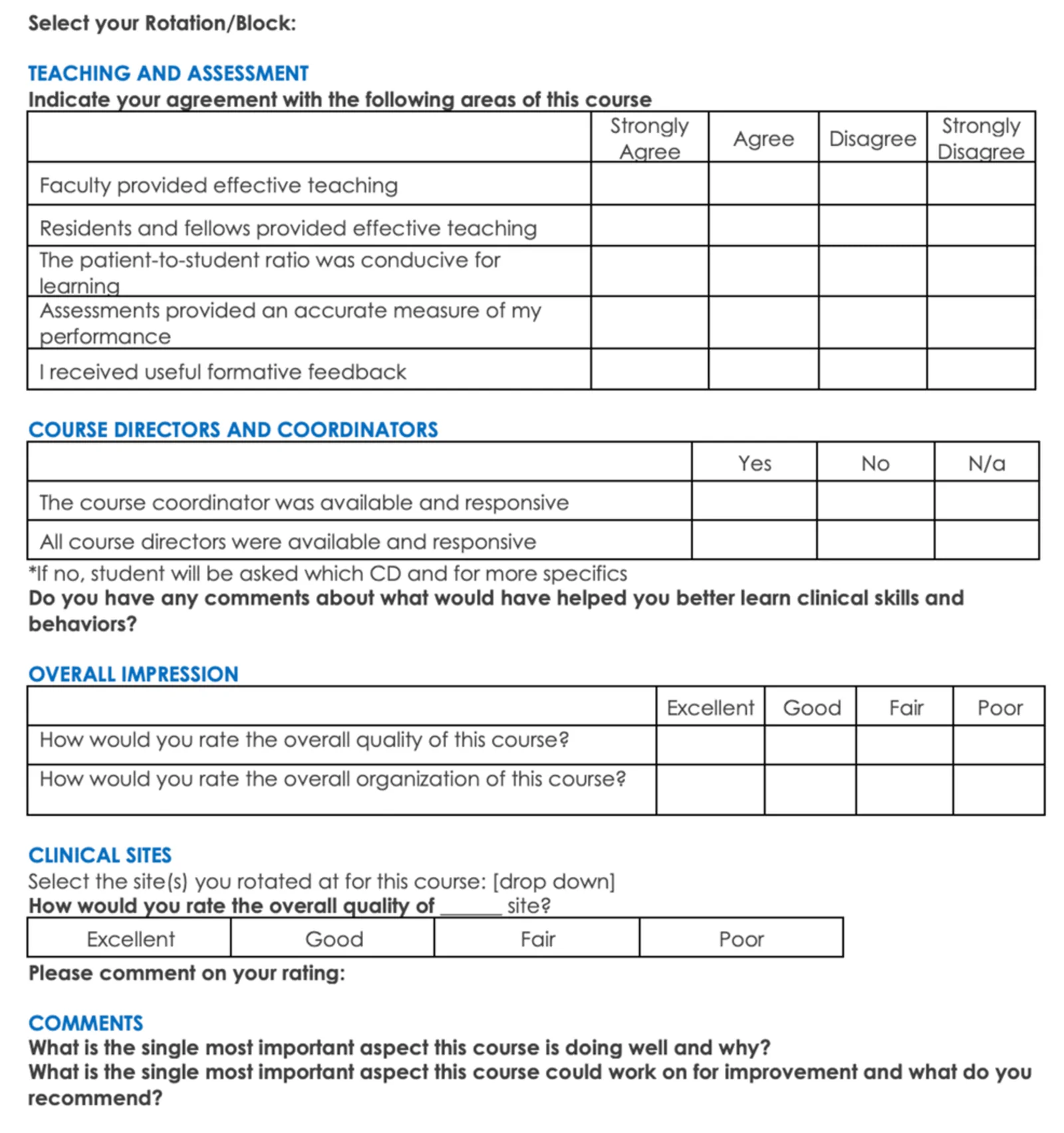
Initial Survey following completion of the University of Utah Sub-Internship.

**Figure 6:**
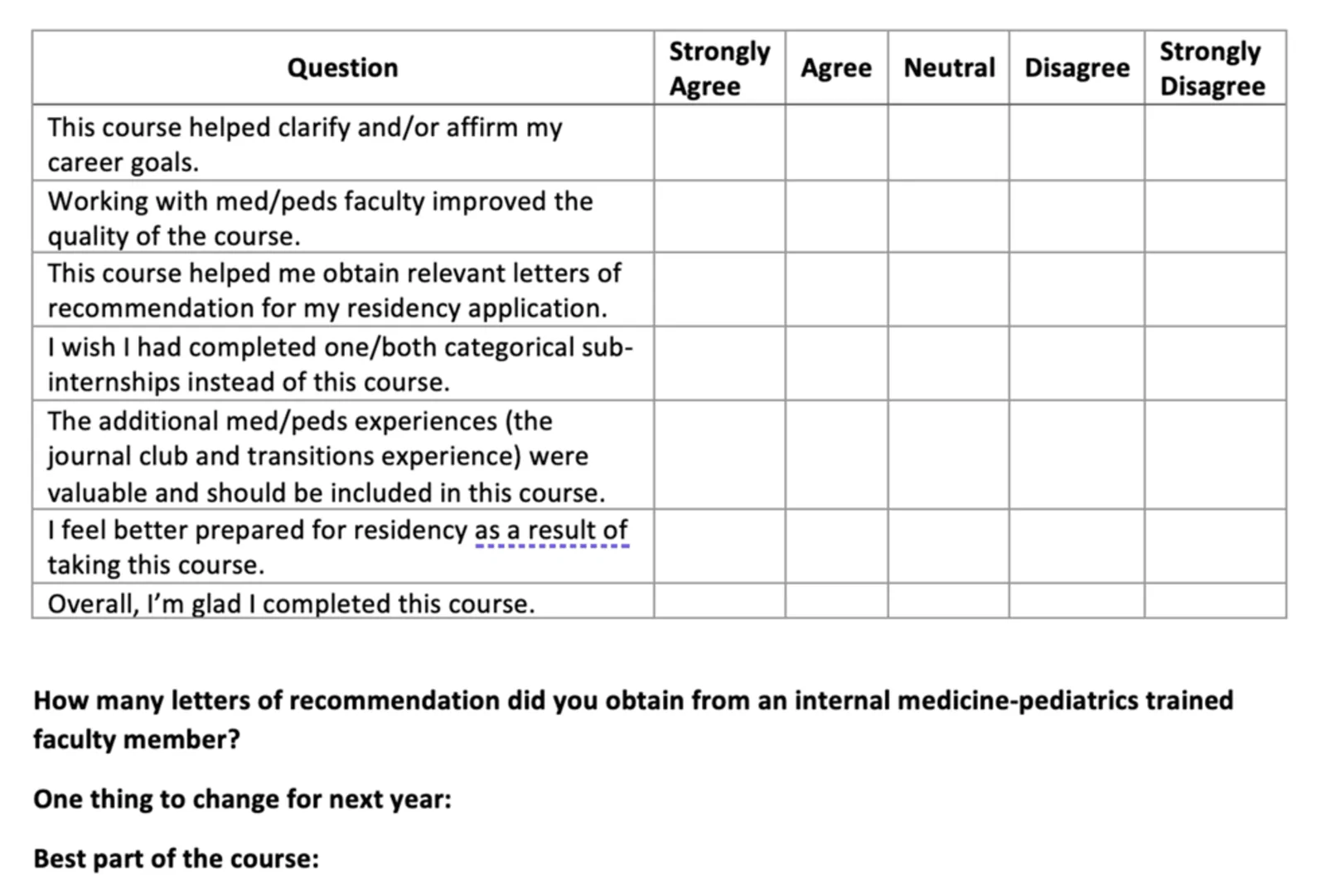
Survey collected 6-months following completion of the University of Utah Sub-Internship.

**Figure 7:**
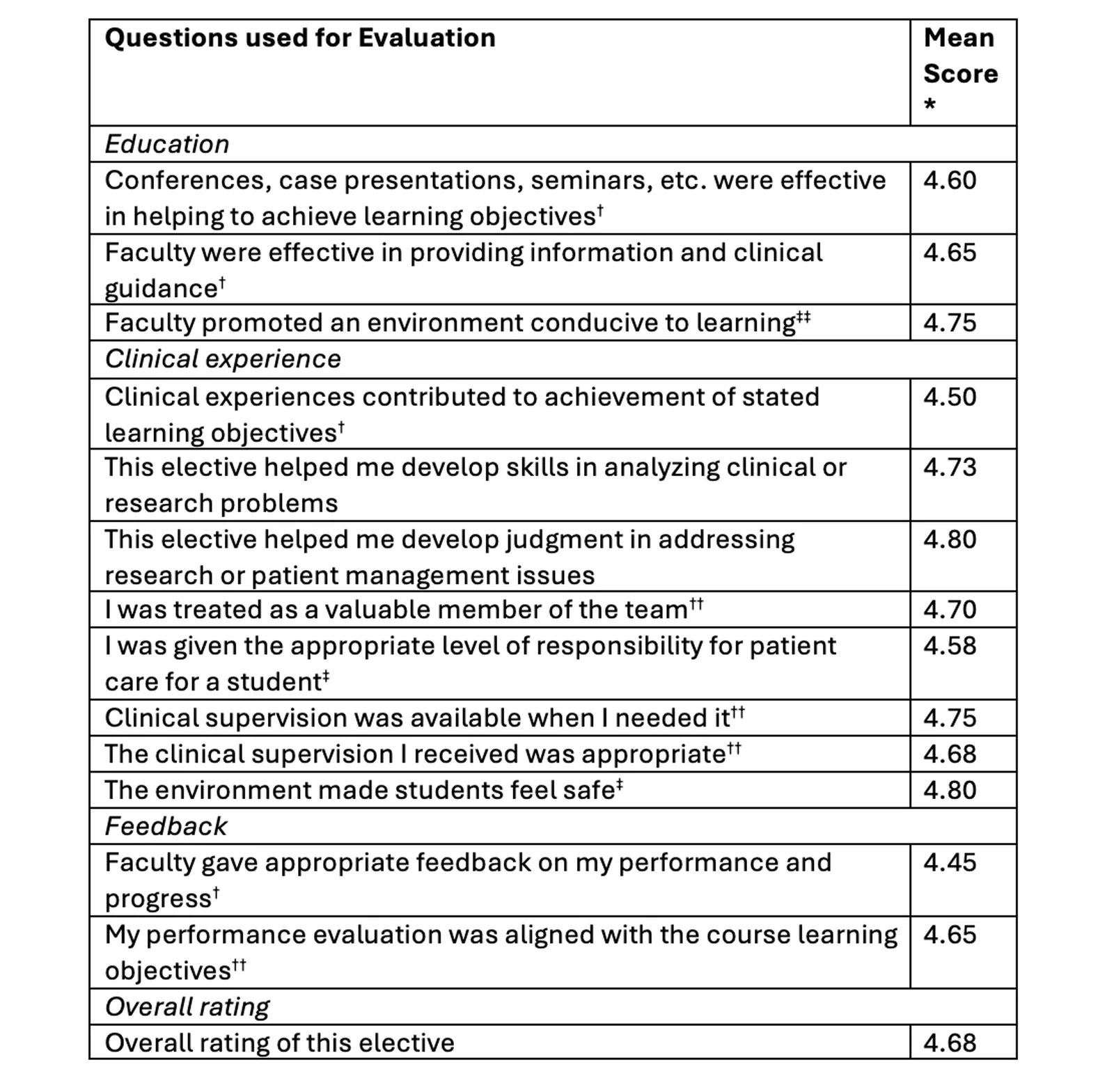
Composite Med-Peds Hospitalist Acting Internship Rotation Evaluation 2012-2020 (N=95) 5-point Likert scale *Students used a 5-point Likert Scale with 1 = “Strongly Disagree,” 2 = “Disagree,” 3 = “Neither Disagree or Agree,” 4 = “Agree,” and 5 = “Strongly Agree,” with the exception of the overall rating of the elective, for which students used a 5-point Likert Scale with 1 = “Poor,” 2 = “Fair,” 3 = “Good,” 4 = “Very Good,” and 5 = “Excellent.” †Questions present only in 2012-2017 evaluations ††Questions present only in 2018-2020 evaluations ‡Questions present only in the 2018 evaluation ‡‡Questions present in all evaluations except for the year 2018 **10 students of the 105 total students who have taken the AI did not complete evaluation

**Figure 8:**
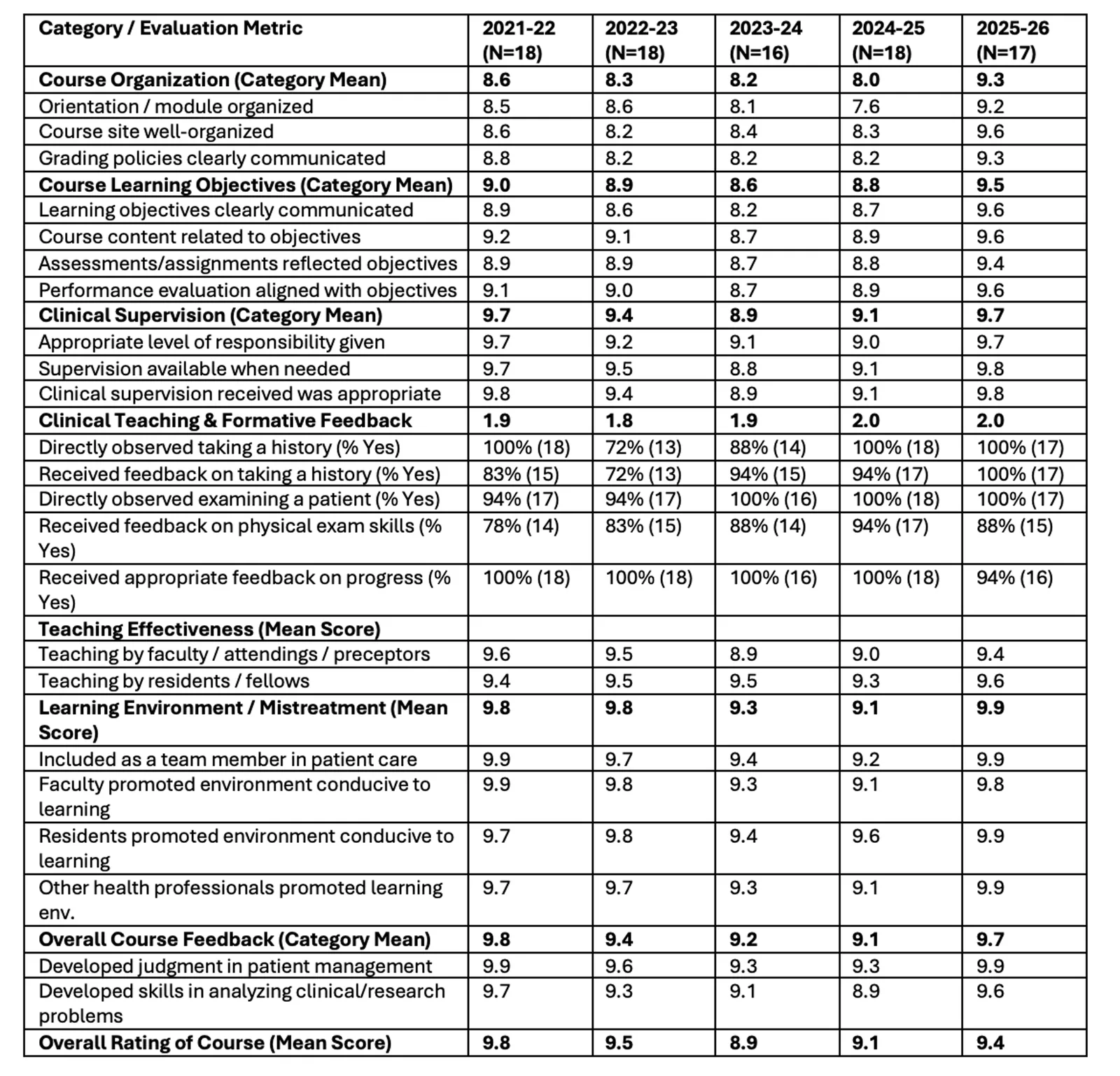
Composite Med-Peds Hospitalist Acting Internship Rotation Evaluation 2021-2026 (N=87)

## Appendices

Appendix A 

Appendix B 

Appendix C

Appendix D

Appendix E 

Appendix F 

## References

[1] Dorsey ER, Jarjoura D, Rutecki GW (2003). Influence of controllable lifestyle on recent trends in specialty choice by US medical students. JAMA.

[2] Him HK, Tim KL, Yvette L (2007). Factors influencing career choices made by medical students, residents, and practicing physicians. BC Med J.

[3] Barzansky B, Walker F (2025). MD-Granting Medical Schools in the US, 2024-2025. JAMA.

[4] Kaminski A, Falls G, Parikh PP (2021). Clerkship experiences during medical school: influence on specialty decision. Med Sci Educ.

[5] Karthik N, Greenfield M, Otteson T (2023). The perceived impact of curricular and non-curricular factors on specialty interests and choice during medical school at a single center in the United States. BMC Med Educ.

[6] Lyss-Lerman P, Teherani A, Aagaard E, Loeser H, Cooke M, Harper GM (2009). What training is needed in the fourth year of medical school? Views of residency program directors. Acad Med.

[7] Angeli A, Ryan J, Palacios C, Al-Eyd G (2025). Factors influencing medical students’ specialty choice: insights to address the evolving primary care gap. Cureus.

[8] Stagg P, Prideaux D, Greenhill J, Sweet L (2012). Are medical students influenced by preceptors in making career choices, and if so how? A systematic review. Rural Remote Health.

[9] Du J, Sathanathan J, Naden G (2009). A surgical career for New Zealand junior doctors? Factors influencing this choice. J New Zealand Med Assoc.

[10] (2026). In Person Med-Peds Elective. https://www.cureus.com/author_guide.

[11] (2026). Medicine/Pediatrics Inpatient Sub-Internship. https://www.childrensmercy.org/professional-education/student-education/medical-student-programs/medical-student-elective--sub-internship-rotations/medicinepediatrics-inpatient-sub-internship/.

[12] Divatia H, Friedland AR (2020). Virtual med-peds: description of the first virtual Med-Peds student elective during COVID-19. Cureus.

[13] Strausser SA, Dopke KM, Groff D, Boehmer S, Olympia RP (2024). Importance of residency applicant factors based on specialty and demographics: a national survey of program directors. BMC Med Educ.

[14] Kremer TR, Kremer MJ, Kremer KP, Mihalic A (2021). Predictors of getting a residency interview: differences by medical specialty. Med Educ.

